# Development of a Potential Probiotic Fresh Cheese Using Two *Lactobacillus salivarius* Strains Isolated from Human Milk

**DOI:** 10.1155/2014/801918

**Published:** 2014-05-29

**Authors:** Nivia Cárdenas, Javier Calzada, Ángela Peirotén, Esther Jiménez, Rosa Escudero, Juan M. Rodríguez, Margarita Medina, Leónides Fernández

**Affiliations:** ^1^Departamento de Nutrición, Bromatología y Tecnología de los Alimentos, Universidad Complutense de Madrid, Ciudad Universitaria, Avenida Puerta de Hierro s/n, 28040 Madrid, Spain; ^2^Probisearch, 28760 Tres Cantos, Spain; ^3^Instituto Nacional de Investigación y Tecnología Agraria y Alimentaria, INIA, Carretera de La Coruña, km. 7.5, 28040 Madrid, Spain

## Abstract

Cheeses have been proposed as a good alternative to other fermented milk products for the delivery of probiotic bacteria to the consumer. The objective of this study was to assess the survival of two *Lactobacillus salivarius* strains (CECT5713 and PS2) isolated from human milk during production and storage of fresh cheese for 28 days at 4°C. The effect of such strains on the volatile compounds profile, texture, and other sensorial properties, including an overall consumer acceptance, was also investigated. Both *L. salivarius* strains remained viable in the cheeses throughout the storage period and a significant reduction in their viable counts was only observed after 21 days. Globally, the addition of the *L. salivarius* strains did not change significantly neither the chemical composition of the cheese nor texture parameters after the storage period, although cheeses manufactured with *L. salivarius* CECT5713 presented significantly higher values of hardness. A total of 59 volatile compounds were identified in the headspace of experimental cheeses, and some *L. salivarius*-associated differences could be identified. All cheeses presented good results of acceptance after the sensory evaluation. Consequently, our results indicated that fresh cheese can be a good vehicle for the two *L. salivarius* strains analyzed in this study.

## 1. Introduction

Among all dairy products, cheese has the highest consumption rate worldwide because of its versatility. Fresh cheeses are usually not or minimally aged, have high moisture content, do not have a rind, and got very mild flavour and a soft and smooth texture. In this category, milk coagulation is due to rennet and/or acid produced from a bacterial culture or other sources such as lemon juice. When bacteria are involved in their manufacture, they also contribute to develop typical flavours, to improve quality, and/or to promote health benefits if they display probiotic properties [1].

Probiotics are defined as “live microorganisms which when administered in adequate amounts confer a health benefit on the host” [2], being* Lactobacillus* and* Bifidobacterium* the most frequently used genera [3]. Yogurt and fermented milks are the most common foods for delivery of probiotic bacteria, but some studies have found that their characteristics may compromise the viability of the probiotic strains [4–6]. Cheese may offer several advantages as a probiotic carrier due to its higher pH and fat content and harder consistency compared to fermented milks [7]. These features provide more protection to probiotics not only during cheese production, ripening, and storage, but also during the passage through the gastrointestinal tract, allowing bacteria to arrive in higher numbers at the target site after ingestion [7].

Several studies have confirmed that human milk is a source of live bacteria, mainly staphylococci and streptococci, but also contains lactic acid bacteria and bifidobacteria [8–11]. The lactobacilli species more frequently isolated from milk samples of healthy women are* Lactobacillus casei*,* Lactobacillus fermentum*,* Lactobacillus gasseri*,* Lactobacillus gastricus*,* Lactobacillus plantarum, Lactobacillu*s* reuteri*,* Lactobacillus salivarius*, and* Lactobacillus vaginalis* [12]. Some lactobacilli isolated from human milk have been characterized and shown to have probiotic potential [13–15]. Specifically,* L. salivarius* CECT5713 that was isolated from human milk and infant feces of a healthy mother-child pair has been shown to have remarkable probiotic potential because it had high rate of survival in simulated gastrointestinal tract conditions and strong adherence to mucus and intestinal cells* in vitro*, stimulated the expression of mucin-encoding genes, and produced antimicrobial compounds [14–17]. More recently, its complete genome has been sequenced [18], and its genetic features, such as proteins potentially involved in human molecular mimetism, may explain its immunomodulatory, anti-inflammatory, and anti-infectious properties [19, 20]. Moreover, its safety and health beneficial effects have been proved in animal models and in human clinical assays [20–23]. More recently,* L. salivarius* PS2 has also been isolated from human milk and preliminary assays have shown similar traits and probiotic potential.

The aim of this work was to evaluate the performance of these two human milk* L. salivarius* strains (CECT5713 and PS2) in fresh cheese in order to develop a probiotic cheese. The survival of these two* L. salivarius *strains in the cheese has been studied as well as their impact on chemical composition, volatile compounds, texture and other organoleptic properties, and overall consumer acceptance of the experimental cheeses.

## 2. Materials and Methods

### 2.1. Starter and Probiotic Organisms

*Lactococcus lactis* ESI153, originally isolated from artisanal raw milk cheese [24], was selected to be used as starter culture.* Lc. lactis* ESI153 cells were grown in M17 (Oxoid, Basinstoke, UK) broth supplemented with 0.5% (wt/vol) glucose (GM17) at 32°C. Before use,* Lc. lactis* ESI153 cells were subcultured (1%) into reconstituted at 11% (wt/vol) and heat-treated (121°C, 5 min) nonfat dry milk (HT-NFDM) and incubated overnight at 32°C.

Freeze-dried cultures of probiotic* L. salivarius* CECT5713 and PS2 were prepared as follows. A fully grown liquid culture on de Man, Rogosa, and Sharpe (MRS) (Oxoid) broth was centrifuged at 10000 ×g for 10 min at 4°C. The cell pellet was washed with 0.85% (wt/vol) NaCl and resuspended in HT-NFDM to one tenth of its original volume. The cell suspension was frozen at −80°C for 12 h in metal trays. Freeze drying was carried out at, first, 0°C for 24 h and, then, at 20°C for 24 h under 1.3 Pa in a Lyph-Lock Stoppering Tray Dryer model 77560 (Labconco Corporation, Kansas City, MO, USA). Freeze-dried cultures containing approximately 10.3 log_10_ colony forming units (cfu)/g were vacuum packaged and stored at 4°C before use.

### 2.2. Experimental Cheese Manufacture

**Figure 1:**
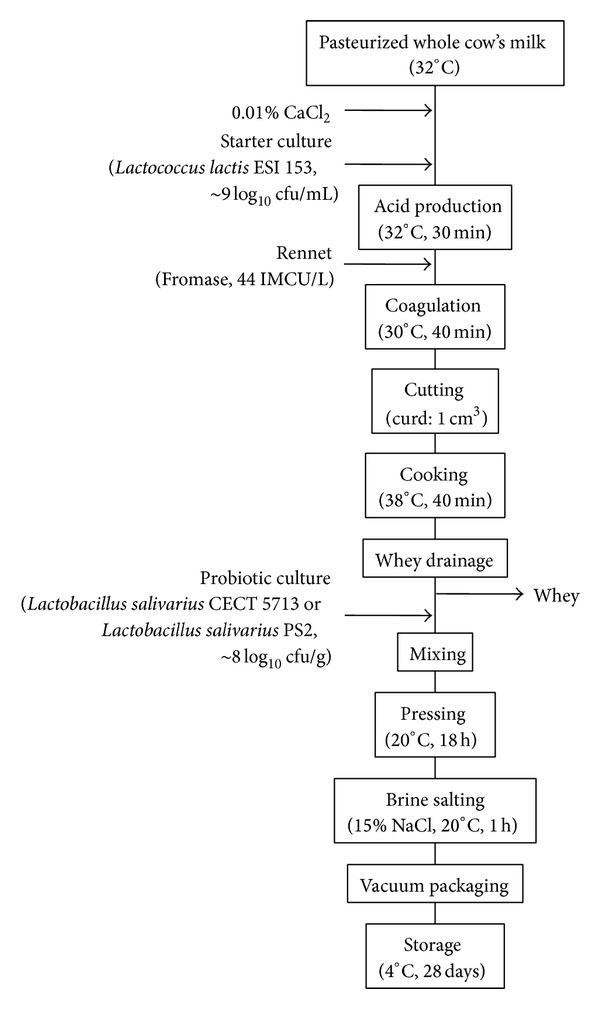
Flowchart of the cheese making process.

Cheeses were made from commercial pasteurized (high temperature short time, HTST) cow's milk (Ganadería Priégola SA, Villanueva del Pardillo, Madrid, Spain) following a laboratory-scale procedure described previously by Rodríguez et al. [25] and Reviriego et al. [26] with some modifications (Figure 1). Briefly, pasteurized milk (1.5 L/vat) at 32°C with 0.01% (wt/vol) CaCl_2_ was inoculated with* Lc. lactis* ESI153 (approximately 9 log_10_ cfu/mL) as starter culture. Rennet (Fromase, 44 IMCU/L; DSM Food Specialities, Seclin Cedex, France) was added to milk 30 min after the inoculation of* Lc. lactis *ESI153. Curds were cut 40 min after rennet addition and heated at 38°C for 40 min. Whey was drained off and freeze-dried* L. salivarius* CECT5713 or PS2 were added to the curd, to reach a final concentration of ~8 log_10_ cfu/g before the curds were distributed into the molds. Control cheese was manufactured at the same conditions with the addition of the equivalent amount of HT-NFDM used as the excipient for freeze drying the lactobacilli strains. Cheeses were pressed for 16 h at room temperature and salted in 15% brine (wt/vol) during 3 h. The resulting cheeses (~190 g) were cut into pieces, which were individually vacuum-packed in Cryovac plastic bags and kept refrigerated at 4°C during 28 days. All cheese manufacturing trials were made in triplicate.

### 2.3. Gross Composition, pH, and Water Activity in Cheese

Cheese samples were analyzed for moisture (ISO 5534/IDF 004:2004), fat (ISO 1735/IDF 005:2004), protein (ISO 8968-1/IDF 020-1:2014), and ash content (AOAC 935.42). The pH value of a cheese slurry prepared by blending 20 g of grated cheese with 12 mL of water [27] was measured with a pH meter (Crison Digit-501). The water activity (*a*_w_) was determined with a Decagon CX-1 hygrometer (Decagon, Pullman, Washington, USA). Determinations were made on triplicate samples.

### 2.4. Viable Bacterial Counts in Cheese

Viability of the* L. salivarius* strains was monitored in cheese samples at 0, 7, 14, 21, and 28 days at 4°C. For this purpose portions (10 g) of cheese were blended with 100 mL of 0.1% (wt/vol) sterile peptone water in a stomacher. Serial dilutions were made also in sterile peptone water and plated following the surface plate technique in appropriate media.* L. salivarius *strains were enumerated on de Man, Rogosa and Sharpe (MRS, Oxoid) agar containing 0.002% (wt/vol) of bromophenol blue after 24 h at 37°C under aerobic conditions.* Lc. lactis* ESI153 was enumerated on M17 (Oxoid) agar supplemented with 0.5% (wt/vol) glucose (GM17) after 24 h at 32°C under aerobic conditions. To confirm their identity, selected colonies were observed by optical microscopy to check their morphology and Gram staining and typed by Random Amplification of Polymorphic DNA (RAPD) using primer OPL5 (5′-ACG CAG GCA C-3′), as described by Ruiz-Barba et al. [28]. Ten randomly chosen isolates sharing the same RAPD profile (only two different RAPD profiles were obtained) were subjected to Pulse Field Gel Electrophoresis (PFGE) after digestion with* Sma*I following the procedure described by Martín et al. [12]. The absence of* Enterobacteriaceae* and* Bacillus cereus* in cheese samples was assessed by pouring onto MacConkey and PEMBA agar (BioMérieux, Marcy l'Etoile, France), respectively, and incubation in aerobically conditions at 37°C for up to 48 h. Bacterial counts were recorded as the cfu/g of cheese and transformed to log_10_ values before statistical analysis.

### 2.5. Isolation of Bacterial DNA and PCR-DGGE Analysis

Cheeses samples (5 g) were homogenized into sodium citrate (50 mL) using a stomacher (260 rpm × 1 min). Then, an aliquot of the mixture (10 mL) was centrifuged at 19,000 ×g during 5 min. The resulting pellet was used for the isolation of total bacterial DNA from each cheese sample following the protocol described previously by Moles et al. [29]. DNA yield was measured using a NanoDrop ND 1000 UV spectrophotometer (Nano-Drop Technologies, Wilmington, Delawere, USA) and was stored at −20°C until PCR DGGE analysis.

Primers U968-GC-f (5′-CGC CCG GGG CGC GCC CCG GGC GGG GCG GGG GCA CGG GGG GAA CGC GAA GAA CCT TAC-3′) and L1401-r (5′-CGG TGT GTA CAA GAC CC-3′) [30] and Lab159f (5′-GGA AAC AGG TGC TAA TAC CG-3′) and Uni-515-GCr (5′-CGC CCG GGG CGC GCC CCG GGC GGG GCG GGG GCA CGG GGG GAT CGT ATT ACC GCG CTG CTG GCA C-3′) [31] were used to amplify V6–V8 regions from 16S rRNA genes on bacterial DNA. The PCR reaction was performed in a total reaction volume of 50 *μ*L containing 5× My Taq Red reaction buffer (Bioline, London, UK), My Taq Red DNA polymerase (Bioline), and 10 g/mL of the isolated DNA. The amplification program was as follows: 95°C for 2 min, 35 cycles of 95°C for 30 s, 56°C for 40 s, 72°C for 60 s, and then 72°C for 5 min. PCR products were stored at −20°C until use.

PCR fragments were separated by denaturing gradient gel electrophoresis (DGGE) using a DCode System (BioRad Laboratories, Inc., Hercules, California, USA) and gels with a linear denaturant gradient of 30 to 50% as described by Martín et al. [32]. A DNA mixture made with equal amounts of amplicons from* L. salivarius* CECT5713 or PS2 and* Lc. lactis* ESI153 was used as a marker.

### 2.6. Analysis of Cheese Texture

Texture profile analysis (TPA) of the cheeses was performed in a texturometer TA-XT2i (Stable Micro Systems Ltd., Surrey, UK). The texturometer was provided with a 0.2 N load cell and a 20 mm diameter probe at a crosshead speed of 5 mm/s to perform a uniaxial compression test in two consecutive compressions. Cheese samples were prepared by cutting 2 cm^3^ cubes, which were kept during 1 h at 25°C before performing the assay. The cheese cube was placed between the two parallel plates and compressed to 50% of its original height sample. TPA parameters (hardness, cohesiveness, adhesiveness, chewiness, gumminess, and springiness) were determined from the TPA two-compression force-time curve with the aid of the Texture Expert for Windows software, version 1.20 (Stable Micro Systems). All measurements were made in triplicate.

### 2.7. Colour Analysis

The colour of cheese samples was determined with a tristimulus colour analyzer (Minolta Chroma Meter CR300, Minolta Corporation, Ramsey, NJ, USA) that measures reflective colours. The measurement was made both on the surface and the core of cheese samples, and the results were expressed using the CIE L*a*b* (CIELAB) space. This three-dimensional model describes all the colours visible to the human eye by means of three spatial coordinates: a central vertical axis that represents lightness (L*) in which values run from 0 (black) to 100 (white); a second perpendicular axis (a*) that represents the red-green channel, where positive values indicate red and negative values indicate green; and the third perpendicular axis (b*) that represents the opponent yellow-blue channel where positive values indicate yellow and negative values indicate blue. Therefore, each colour can be represented as a point in a three-dimensional space defined by its three parameters L*, a*, and b*. All measurements were made in triplicate.

### 2.8. Analysis of Volatile Compounds

Cheese samples were wrapped in aluminium foil, vacuum-packed in Cryovac plastic bags, and frozen at −80°C until analysis. Volatile compounds were extracted by headspace solid-phase microextraction (SPME) and, then, analysed by gas chromatography-mass spectrometry GC-MS (HP6890-MSD HP 5973, Agilent, Palo Alto, CA, USA) according to the procedure described by Lee et al. [33]. Cheese samples (10 g) were homogenized with anhydrous sodium sulphate (20 g) and 20 *μ*L of an aqueous solution containing cyclohexanone (1058 ppm) and camphor (1040 ppm) as internal standards using a mechanical grinder. Then, 5 g of this mixture was weighed in a 40 mL glass vial that was sealed with a polytetrafluoroethylene (PTFE) faced silicone septum. Volatile compounds were isolated using a SPME manual holder equipped with a 2 cm × 50/30 *μ*m Stable Flex Divinylbenzene/Carboxen/Polydimethylsiloxane (DVB/CAR/PDMS) coated fibre (Supelco, Bellefonte, Pennsylvania, USA). Vials were equilibrated in a thermostatic bath at 37°C for 20 min before the fiber was inserted through the PTFE septum for headspace extraction. The fiber was exposed to the headspace for 30 min, and then it was inserted into the GC injection port for desorption (260°C/10 min in splitless mode).

Separation of volatile compounds was performed on a Zebron ZB-WAX plus (60 m × 0.25 mm × 0.50 *μ*m) capillary column coated with 100% polyethylene glycol (Phenomenex, Torrance, California, USA). For chromatographic separation, the temperature was maintained at 40°C for 7 min, increased from 40°C to 90°C at a rate of 2°C/min, from 90°C to 150°C at a rate of 3°C/min, from 150°C to 240°C at a rate of 9°C/min and, finally, held at 240°C for 8 min. Detection was performed with the mass selective detector operating in the scan mode, collecting data at a rate of 5.16 scans/s over a range of 33–300 m/z at ionization energy of 70 eV. Identification of volatile compounds was based on comparison of spectra using the Wiley 275 Library (Wiley and Sons Inc., New York, USA). Relative abundances of compounds were expressed as percentages of their peak areas to the cyclohexanone peak area. Samples were tested in duplicate.

### 2.9. Sensory Evaluation

The sensory evaluation of cheeses was done by panellists (staff and students of the Department of Food Science and Nutrition, Universidad Complutense de Madrid) who were familiar with sensory evaluation techniques. The evaluation was performed in individual booths under controlled conditions of environment and light.

Representative cheese samples (~20 g) after 28 d of storage at 4°C were equilibrated at room temperature and presented to the tester in disposable plastic containers, except for odour assessment for which samples were presented in closed glass flasks. Samples were codified with random 3 digit numbers following a completely randomized block design. Cheese portions from replications of the same batch were mixed, so a representative sample was presented to the panellists.

Initially, 30 tester semitrained panellists participated in a triangle test to determine if potential probiotic cheeses containing lactobacilli differ in any aspect from the control cheese. Significant differences were determined using the method of Roessler et al. [34]. Later on, 18 selected trained panellists were asked to perform a descriptive test for a number of specific descriptors clustered in groups related to odour (buttery, cow, fermented milk, floral, fruity, lawn, rancid, and vinegar), flavour and taste (aftertaste, astringent, bitter, cow, fermented milk, fruity, salty, and vinegar), and texture and appearance (adhesive, bright, colour (white or yellow), creamy, friable, hardness, moist, springy, and smooth) and to score for the overall impression using a 10-point intensity scale.

### 2.10. Statistical Analysis

The influence of the addition of* L. salivarius* CECT5713 and PS2 to cheeses was analyzed by one way analysis of variance (ANOVA). Tukey's multiple range tests were applied to determine differences among the cheeses. Differences were considered significant at *P* < 0.05. StatGraphics Centurion XVI version 16.1.15 (Statpoint Technologies Inc., Warrenton, Virginia, USA) was used to perform these analyses.

## 3. Results

### 3.1. Gross Composition, pH, and Water Activity

The mean composition of control cheeses without the addition of probiotic bacteria was 24.5% (wt/wt) fat, 17.8% (wt/wt) protein, and 3.1% (wt/wt) ash after 28 days of storage at 4°C (Table 1). Chemical composition of cheeses manufactured with* L. salivarius* CECT5713 or PS2 was similar; slightly higher moisture and lower fat and protein contents were found compared to control cheeses, although the difference was statistically significant only for the protein content. The presence of lactobacilli in cheeses was associated with a lower final pH compared to control cheeses, but it did not modify the *a*_w_ of the final product (0.96-0.97).

### 3.2. Viability of* L. salivarius* during Cheese Storage

**Figure 2:**
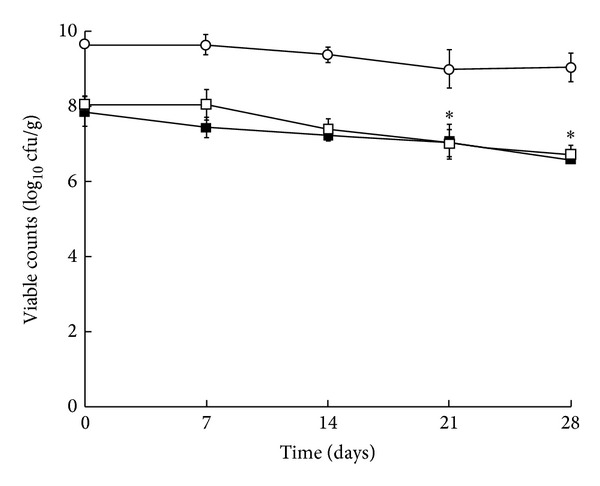
Viable counts (log_10_ cfu/g) of starter* Lc. lactis* ESI153 (○) and* L. salivarius* CECT5713 (□) and PS2 (■) in fresh cheese during storage at 4°C.

The growth of* L. salivarius* CECT5713 and PS2 in MRS agar containing bromophenol produced large and blue colonies, while in the same conditions* Lc. lactis* ESI153 colonies were small and white. Initial viable counts in MRS agar of* L. salivarius *CECT5713 and PS2, according to the morphology of the colony, were 8.1 and 7.9 log_10_ cfu/g, respectively (Figure 2). Both* L. salivarius* strains remained viable in the cheeses after 28 days at 4°C (Figure 2). The probiotic counts decreased during storage, but the reduction in viable counts was significant only after 21 days. Final concentrations of* L. salivarius* CECT5713 andPS2 were 6.7 and 6.6 log_10_ cfu/g, respectively, representing about a 1.3 log_10_-unit reduction at the end of 28-day storage. The identity of selected colonies isolated at the end of the storage period was confirmed by RAPD and PFGE (results not shown). On the other hand, initial viable counts of the starter culture* Lc. lactis* ESI153 were higher (about 9 log_10_ cfu/g in all cheese samples) and remained fairly constant along the storage period. Bacterial growth was not detected on PEMBA and MacConkey agar plates, confirming the absence of contamination with* B. cereus *and* Enterobacteriaceae*.

### 3.3. PCR DGGE Analysis

**Figure 3:**
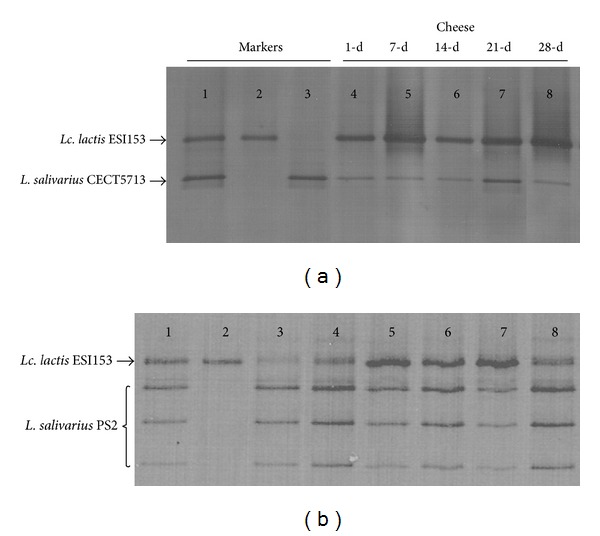
DGGE profiles of 16S rRNA gene V6–V8 regions obtained from samples of cheese manufactured with* L. salivarius* CECT5713 using universal primers U968-GC-f and L1401-r (a) and* L. salivarius* PS2 using* Lactobacillus*-specific primers Lab159f and Uni-515-GCr (b) during storage at 4°C. (a): lane 1: marker (*L. salivarius* CECT5713 and* Lc. lactis* ESI153); lane 2:* Lc. lactis* ESI153; lane 3:* L. salivarius* CECT5713; lane 4: 1-day cheese; lane 5: 7-day cheese; lane 6: 14-day cheese; lane 7; 21-day cheese; lane 8: 28-day cheese. (b) Lane 1: marker (*L. salivarius* PS2 and* Lc. lactis* ESI153); lane 2:* Lc. lactis* ESI153; lane 3:* L. salivarius* PS2; lane 4: 1-day cheese; lane 5: 7-day cheese; lane 6: 14-day cheese; lane 7; 21-day cheese; lane 8: 28-day cheese.

DGGE analysis was also performed in order to check the bacterial diversity and to confirm the presence of the probiotic strains' DNA in the cheese samples (Figure 3). The amplification of the V6–V8 variable region of the 16S rRNA gene of* L. salivarius* CECT5713 and* Lc. lactis* ESI153 using the primers U968-GC-f and L1401-r resulted in a single fragment differing in size for each bacterial species (Figure 3(a)). The same primer pair did not amplify any fragment when* L. salivarius* PS2 DNA was used as template. However, amplification of one fragment corresponding to the V6–V8 variable region of the 16S rRNA gene of* L. salivarius* PS2 was successful when* Lactobacillus*-specific primers (Lab159f and Uni-515-GCr) were used (Figure 3(b)). The DGGE profile obtained from* L. salivarius* PS2 using this pair of primers comprised 3 dominant bands and it was different from the one obtained for* Lc. lactis* ESI153.

The DGGE profiles of cheese samples analyzed and the ladders constructed in this study (with amplicons obtained from pure cultures of* Lc. lactis* ESI153 and the corresponding* L. salivarius* strain), using universal or* Lactobacillus*-specific primers, were identical (Figure 3). This result indicates that the inoculated strains were the predominant in the respective cheeses during storage.

### 3.4. Textural Analysis of Cheeses

Texture parameters at the end of the cheese storage were similar in control and* L. salivarius*-containing cheeses (Table 2). No significant differences were observed in cohesiveness, adhesiveness, springiness, gumminess, and chewiness values. Globally, cheeses had a crumbly and brittle texture requiring only a relatively small load to fracture. However, cheeses manufactured with* L. salivarius* CECT5713 presented significantly higher values of hardness compared to control cheeses and cheeses containing* L. salivarius* PS2.

### 3.5. Colour Analysis

The colour was measured both on the surface and the core of cheese samples by tristimulus reflectance measurement (Table 3). In general, all samples had high lightness (L* ~ 92 to 94), indicating no differences in the mechanical openings exhibited by the three cheeses, low yellow (b* ~ 11 to 12), and very low green (a* ~ −1 to −2) colour. Globally, the lightness value was lower in the surface than in the interior, possibly reflecting a closer structure in the surface and more open pores in the interior. The opposite was observed for b* parameter, indicating more yellowness in the surface. The surface of cheeses elaborated with* L. salivarius* CECT5713 was whiter and had less intense green colour than the others (Table 3).

### 3.6. Volatile Analysis

A total of 59 volatile compounds were identified in the headspace of experimental cheeses, including aldehydes, ketones, alcohols, esters, alkanes, and carboxylic and fatty acids (Table 4). All cheese samples presented high relative abundance of the alcohols 3-methyl-1-butanol and ethanol as well as of acetic, butanoic, and hexanoic acids, although significant differences were observed only for acetic acid that had higher abundance in cheeses containing* L. salivarius* CECT5713. Aldehyde 3-methylbutanal, ketone 2-heptanone, and alkane 2,4-dimethylheptane were present at statistically significant lower levels in cheeses manufactured with* L. salivarius* than in control cheese. Level of 2-propanone was higher in cheese made with* L. salivarius* PS2 than in the other two cheeses.

### 3.7. Sensory Evaluation

The results of the triangle test to evaluate differences in sensory properties indicated that significant differences were detected at the end of the storage. The panellists could appreciate significant variations between control cheese and cheese manufactured with* L. salivarius *CECT5713 (*P* = 0.018) or PS2 (*P* = 0.002).

**Figure 4:**
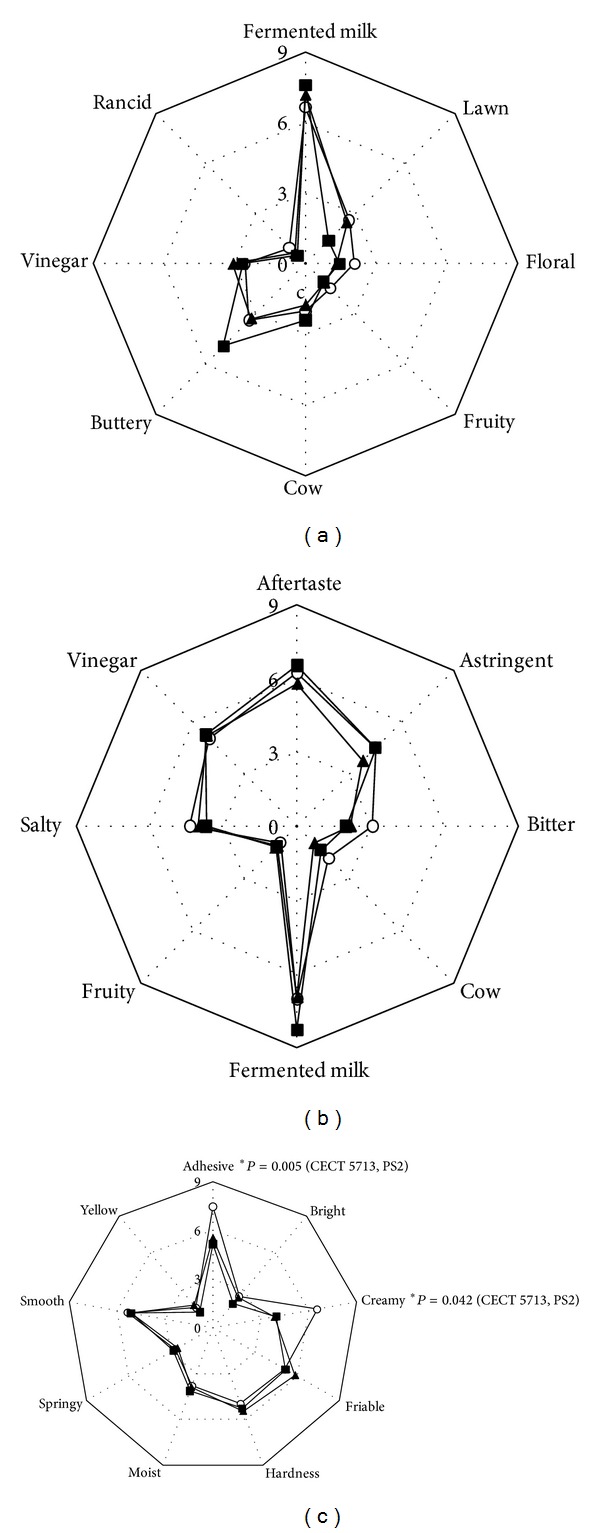
Graphical charts of the sensory profile of control cheese (○) and cheeses containing* L. salivarius* CECT5713 (■) and PS2 (▲) after 28 days at 4°C. (a) Odour related attributes. (b) Flavour and taste related attributes. (c) Texture and appearance descriptors.

Trained panellists performed a quantitative descriptive analysis using attributes describing odour, flavour, taste, texture, and appearance of cheeses after 28 days of storage at 4°C (Figure 4). Globally, the descriptors that obtained the highest scores were fermented milk taste and smell and butter-like smell. Among all attributes, only the intensity of adhesiveness and creaminess in control cheese was statistically higher than that of the cheeses containing* L. salivarius* CECT5713 or PS2. However, these differences did not have any statistically significant effect on the overall quality of the cheeses (*P* < 0.05), and all cheese samples presented good results of acceptance after 28 days of storage at 4°C. In average, the acceptance level of odour, flavour, appearance, and texture, as well as the global score, was up to 6 (on a 0–10 numeric rating scale) in the three types of cheese.

## 4. Discussion

Cheese has been considered as an excellent alternative to fermented milk and yogurts as a food vehicle for probiotic delivery. Its buffering capacity is one of its advantages because it protects probiotics against the highly acidic stomach environment. The structure of the gel and its high fat content and solid consistency also add to the probiotic protection [35, 36]. Several studies have demonstrated that cheese is an excellent carrier for probiotic bacteria, including fresh and Cheddar cheese varieties [5, 27, 36–38]. However, variable results have been obtained with different probiotic strains and each strain should be tested individually. Therefore, the objective of this study was to check the viability of two lactobacilli strains isolated from human milk after their incorporation to cheese curds and to test their impact in the final product.

Theoretically, the probiotic bacteria could be added either directly to milk and/or incorporated at a later stage during the manufacture of cheese. Ong et al. [36] manufactured probiotic Cheddar cheese containing different combinations of six probiotic* Lactobacillus* and* Bifidobacterium* strains which were cocultured with the cheese starter culture. They reported some loss of probiotic lactobacilli and bifidobacteria in whey (about 6-7 log_10_ cfu/g), but final counts in all fresh cheeses were high and acceptable (8-9 log_10_ cfu/g). In preliminary trials, following our procedure,* L. salivarius* CECT5713 and PS2 were also cocultured with the starter culture, but only a small amount of probiotic was retained in the curd, resulting in a high loss of lactobacilli in the cheese whey (results not shown). Notably, the addition of the probiotic lactobacilli to the curds after whey drainage, when most of the whey had been removed and before molding, resulted in improved retention of lactobacilli in the cheese.

In any probiotic food, in order to have industrial application and to exert its health benefit to the host, the incorporated probiotic strain must maintain its viability during the manufacture, through the shelf life of the product and up to the time of consumption. In the present study, both* L. salivarius* strains remained viable in the experimental cheeses after 28 days at 4°C. In addition, the hygienic quality of the final product was adequate and growth of any other bacteria was not detected in the culture media used. Antibacterial properties against pathogenic bacteria have been reported for* L. salivarius* CECT5713 due to the production of antimicrobial compounds such as lactate, acetate, and hydrogen peroxide [14]. This strain also harbors a bacteriocin cluster located in a megaplasmid that contains several genes that would allow the biosynthesis of several bacteriocins, but a deletion at the beginning of the regulatory system results in the absence of any bacteriocin production [19].

A minimum probiotic daily dose of 10^8^-10^9^ cfu has been recommended in processed foods in order to exert their beneficial effects [35, 39]. This would be equivalent to a daily intake of 100 g of product containing 10^6^-10^7^ cfu/g. The results obtained in this study show that the counts of* L. salivarius* CECT5713 and PS2 in cheeses were always in the range of this recommended level. Therefore, they would satisfy this criteria established for a probiotic food. Furthermore, the presence of these potentially probiotic bacteria did not interfere with the performance of starter lactococci, as it has been described by other authors [27].

Another challenge associated with the addition of probiotic bacteria during cheese manufacturing is to maintain the characteristics of the cheese. Actually, consumers demand the addition of probiotic cultures to many foods, including cheese, but a primary consideration is that the sensory properties, especially taste, of any probiotic food should be appealing [40]. The addition of certain levels of any viable bacteria, and their enzymes, to cheese most probably will contribute to glycolysis, proteolysis, and lipolysis processes that take place during manufacture and cheese ripening and contribute to the organoleptic properties of the final product [41]. In order to maintain an adequate organoleptic quality, probiotic bacteria must not adversely affect cheese composition, texture, flavour, and final acceptance. The addition of probiotic* L. salivarius* CECT5713 and PS2 did not result in a substantial change of the fresh cheese composition, which was within the gross chemical composition of this type of cheeses.

A slight (but not statistically significant) increase in moisture content of cheeses containing lactobacilli compared to control cheese was noted, although *a*_w_ values remained unchanged. These differences may be related, at least in part, to several factors during cheese manufacture that exert a great influence on moisture retention in the curd such as cutting intensity, final size of the curds, or curd manipulation [42]. On the other hand, exopolysaccharide- (EPS-) producing lactic acid bacteria have been reported to increase moisture retention in cheese [43–45] and could improve the texture of reduced-fat cheese that tends to be tough and rubbery. Two gene clusters for EPS biosynthesis have been described in* L. salivarius* strains, although it has been reported that the level of production of EPS does not correlate with the presence of these clusters, depends on the available carbohydrate, and is highly strain-dependent [46]. However, at present it is not known if* L. salivarius* CECT5713 and PS2 are EPS-producing strains. Another tentative explanation may involve microbial dynamics and metabolism. It has been reported that cheese microbiota and its metabolic activity may confound the effect of moisture on *a*_w_ [47]. Changes in the type and concentration of low molecular weight soluble compounds, such as an increase in lactate, free fatty acids, amino acids, and, even, very small peptides, might decrease the value of *a*_w_, although this effect is usually more pronounced for ripened cheeses [47, 48].

Among the texture parameters analyzed in this work, instrumental methods only detected differences in hardness, which was higher in cheeses containing* L. salivarius* CECT5713 than in control cheeses and those manufactured with* L. salivarius* PS2. However, this difference was not perceived by panellists during the descriptive test, indicating that it did not have a relevant impact in the sensory quality and acceptance of the cheese. On the contrary, the panellists identified both cheeses containing probiotic lactobacilli as having lower adhesiveness than the control cheese, although the texture profile analysis did not reveal a statistically significant difference. Reduction in adhesiveness in cheeses made with an EPS-producing* Streptococcus thermophilus* culture has been related to the production and liberation of EPS [49, 50]. Also, a higher perception of creaminess in control cheese was reported in contrast to cheeses manufactured with* L. salivarius* CECT5713 and PS2. Creaminess is often related to a high fat level and the presence of fat globules, in agreement with the slightly higher fat content of control cheese, although sensorial discrimination of fat levels in solid foods is more difficult than in liquid products [51]. Regarding colour, small although statistically significant differences were also detected when using instrumental methods of analysis, but they were not perceived by the trained panellists. This indicates that the presence of probiotic lactobacilli did not disturb the distinctive white colour of fresh cheese.

Following the general component balance theory, cheese flavour is the result of a synergistic effect of the appropriate and balanced blend of various flavour compounds produced from proteins, lipids, and lactose through numerous biochemical reactions involving enzymes from milk, rennet, starter cultures, secondary cheese microbiota, and, even, spontaneous reactions [52]. The volatile composition of the cheese made with both* L. salivarius* strains was not qualitatively different from that of the control cheese, and only a few quantitative differences were observed. The main change detected was a higher acetic acid concentration in the cheeses containing* L. salivarius *CECT5713 and PS2, probably related to higher lactose degradation during cheese storage which will explain a lower final pH in the probiotic cheese. However, these differences did not impact the sensory perception given by the panellists or the global acceptance of the cheeses manufactured with* L. salivarius* CECT5713 and PS2, as it has been described with other probiotic strains by other authors [36–38, 49, 50, 53].

## 5. Conclusion

The results of the present study demonstrate that* L. salivarius* CECT5713 and PS2 incorporated into fresh cheese survived at adequate levels during a 28-day storage at 4°C. The presence of the lactobacilli did not interfere with normal growth of starter culture and did not modify significantly the composition and organoleptic properties of the probiotic cheeses containing* L. salivarius* strains that had good acceptance by trained panellists.

## Figures and Tables

**Table 1:** Chemical composition, pH, and water activity of control cheese and cheeses using *L*. *salivarius* CECT5713 or PS2 after 28 days of storage at 4°C*.

	Cheese			*P* value**
	Control	*L*. *salivarius* CECT5713	*L*. *salivarius* PS2	
Moisture (%, wt/wt)	52.48 ± 2.14	56.17 ± 1.95	56.62 ± 1.96	0.086
Fat (%, wt/wt)	24.47 ± 1.30	20.88 ± 1.84	22.37 ± 1.27	0.067
Protein (%, wt/wt)	17.75 ± 0.80^a^	14.94 ± 0.79^b^	15.43 ± 0.85^b^	0.012
Ash (%, wt/wt)	3.10 ± 0.11	3.28 ± 0.45	2.88 ± 0.10	0.278
pH	4.86 ± 0.13	4.71 ± 0.03	4.75 ± 0.11	0.287
*a*_*w*_	0.96 ± 0.01	0.96 ± 0.01	0.97 ± 0.01	0.398

*Results are expressed as mean ± standard deviation values of triplicate samples; **one-way ANOVA to determine differences on chemical composition, pH, and *a*_*w*_ between cheeses. ^a,b^Mean values within the same row followed by different letter were significantly different when compared using the Tukey's test.

**Table 2:** Texture profile analysis of control and experimental cheeses using *L*. *salivarius* CECT5713 or PS2 after 28 days of storage at 4°C*.

Parameter	Cheese			*P* value**
	Control	*L*. *salivarius* CECT5713	*L*. *salivarius* PS2	
Hardness (N)	21.00 ± 0.71^a^	24.42 ± 0.97^b^	21.27 ± 0.65^a^	0.001
Cohesiveness	0.15 ± 0.03	0.15 ± 0.01	0.14 ± 0.01	0.662
Adhesiveness (N s)	−0.57 ± 0.30	−0.77 ± 0.09	−0.57 ± 0.04	0.236
Springiness (m)	0.0048 ± 0.0015	0.0043 ± 0.0017	0.0048 ± 0.0010	0.851
Gumminess (N)	3.22 ± 0.64	3.61 ± 0.23	2.99 ± 0.29	0.171
Chewiness (N m)	0.015 ± 0.004	0.014 ± 0.005	0.013 ± 0.001	0.927

*Texture parameters are expressed as mean ± standard deviation values of quadruplicate measurements in triplicate samples; **one-way ANOVA to determine differences on texture parameters between cheeses. ^a,b^Mean values within the same row followed by different letter were significantly different when compared using the Tukey's test.

**Table 3:** Colour parameters (L*, a*, and b*) of control and experimental cheeses manufactured with *L*. *salivarius* CECT5713 or PS2 after 28 days of storage at 4°C^†^.

Parameter	Cheese			*P* value^††^
	Control	*L*. *salivarius* CECT5713	*L*. *salivarius* PS2	
Surface				
L*	92.20 ± 0.90	92.67 ± 0.56	92.41 ± 0.12	0.664
a*	−1.58 ± 0.02^a^	−1.16 ± 0.04^b^	−1.33 ± 0.11^c^	0.001
b*	11.66 ± 0.41^a^	10.74 ± 0.16^b^	11.92 ± 0.26^a^	0.006
Core				
L*	93.13 ± 0.57	93.29 ± 0.08	93.81 ± 0.46	0.202
a*	−1.44 ± 0.09^a^	−1.48 ± 0.12^ab^	−1.70 ± 0.04^b^	0.024
b*	10.75 ± 0.17	10.69 ± 0.21	10.92 ± 0.34	0.521

^†^Colour parameters are expressed as mean ± standard deviation values of quadruplicate measurements in triplicate samples; ^††^one-way ANOVA to determine differences on colour parameters between cheeses. ^a,b,c^Means values within the same row followed by different letter were significantly different when compared using the Tukey's test.

**Table 4:** Volatile compounds in control cheese and cheeses containing *L*. *salivarius* CECT5713 or PS2 after 28 days at 4°C*.

Volatile compound	RT**	Cheese			*P* value***
		Control	*L. salivarius* CECT5713	*L. salivarius* PS2	
Aldehydes					
3-Methylbutanal	12.42	2.82 ± 0.15^a^	0.98 ± 0.14^b^	1.62 ± 0.25^c^	0.000
Hexanal	23.21	0.64 ± 0.24	0.50 ± 0.07	0.56 ± 0.06	0.552
Ketones					
2-Butanone	8.01	3.66 ± 0.28	2.25 ± 0.39	2.64 ± 0.86	0.101
2-Propanone	11.58	16.85 ± 1.99^a^	15.00 ± 1.36^a^	21.39 ± 1.08^b^	0.001
2-Heptanone	30.78	4.02 ± 0.16^a^	1.89 ± 0.29^b^	2.09 ± 0.09^b^	0.000
3-Hydroxy-2-butanone	38.08	2.14 ± 0.36	1.91 ± 1.46	0.98 ± 0.31	0.413
Alcohols					
Ethanol	13.76	45.05 ± 2.86	47.95 ± 3.96	50.79 ± 3.01	0.212
3-Methyl-1-butanol	32.89	348.48 ± 6.49	358.80 ± 12.98	368.84 ± 20.33	0.375
2-Furanmethanol	55.59	0.87 ± 0.06	0.11 ± 0.05	0.52 ± 0.49	0.217
3-Methyl-3-buten-1-ol	35.71	1.04 ± 0.03	1.04 ± 0.10	1.18 ± 0.14	0.138
Esters					
Ethyl acetate	10.93	0.71 ± 0.01	0.65 ± 0.20	0.69 ± 0.15	0.905
Ethyl butanoate	20.02	0.55 ± 0.19	1.00 ± 0.22	1.06 ± 0.21	0.110
Ethyl hexanoate	34.41	0.34 ± 0.03	0.51 ± 0.11	0.54 ± 0.15	0.221
Alkanes					
2-Methylpentane	4.33	0.31 ± 0.04	0.35 ± 0.06	0.50 ± 0.17	0.200
3-Methylpentane	4.44	0.25 ± 0.02	0.34 ± 0.06	0.47 ± 0.15	0.073
Hexane	4.51	1.82 ± 0.36	1.82 ± 0.46	1.80 ± 0.64	0.998
Heptane	5.40	0.72 ± 0.01	0.65 ± 0.19	0.40 ± 0.09	0.064
2,4-Dimethylheptane	7.55	1.32 ± 0.05^a^	0.40 ± 0.09^b^	0.40 ± 0.15^b^	0.001
4-Methyloctane	9.30	0.83 ± 0.04	0.20 ± 0.03	0.28 ± 0.18	0.113
Carboxylic and fatty acids					
Acetic acid	46.78	22.76 ± 1.02^a^	42.61 ± 2.37^b^	33.53 ± 8.62^ab^	0.016
Propanoic acid	50.93	0.07 ± 0.00	0.08 ± 0.00	0.08 ± 0.00	
Butanoic acid	54.47	20.36 ± 1.80	28.23 ± 2.99	26.15 ± 6.26	0.209
Hexanoic acid	59.58	14.82 ± 0.99	22.49 ± 3.51	20.04 ± 4.14	0.110
Octanoic acid	62.89	7.42 ± 2.30	13.80 ± 3.86	10.85 ± 2.04	0.133
Heptanoic acid	61.33	0.48 ± 0.01	0.62 ± 0.09	1.08 ± 0.44	0.083
Nonanoic acid	64.46	1.35 ± 0.19	0.73 ± 0.26	6.58 ± 8.90	0.362
Decanoic acid	66.21	3.15 ± 1.89	5.66 ± 3.18	3.29 ± 1.27	0.401
Others					
D-Limonene	31.78	0.74 ± 0.02	1.18 ± 0.13	0.72 ± 0.47	0.163

*Relative abundance of compounds was expressed as percentages of their peak areas to the cyclohexanone peak area; **RT: retention time; ***one-way ANOVA to determine differences on the relative abundance of volatile compounds between cheeses. ^a,b,c^Mean values within the same row followed by different letter were significantly different when compared using the Tukey's test.
